# Inflammatory markers and risk of cardiovascular mortality in relation to diabetes status in the HUNT study

**DOI:** 10.1038/s41598-021-94995-8

**Published:** 2021-08-02

**Authors:** Lena Løfblad, Gunhild Garmo Hov, Arne Åsberg, Vibeke Videm

**Affiliations:** 1 Department of Clinical Chemistry, St. Olavs University Hospital, Trondheim, Norway; 2 Department of Clinical and Molecular Medicine, NTNU—Norwegian University of Science and Technology, Trondheim, Norway; 3 Department of Immunology and Transfusion Medicine, St. Olavs University Hospital, Trondheim, Norway

**Keywords:** Diabetes, Cardiovascular diseases, Prognostic markers, Epidemiology, Risk factors

## Abstract

Inflammatory markers have been associated with increased risk of cardiovascular mortality in general populations. We assessed whether these associations differ by diabetes status. From a population-based cohort study (n = 62,237) we included all participants with diabetes (n = 1753) and a control group without diabetes (n = 1818). Cox regression models were used to estimate hazard ratios (HRs) with 95% confidence intervals (CI) for possible associations with cardiovascular mortality of 4 different inflammatory markers; C-reactive protein (CRP), calprotectin, neopterin and lactoferrin. During a median follow-up of 13.9 years, 728 (20.4%) died from cardiovascular disease (CVD). After adjustment for age, sex and diabetes, the associations of all inflammatory markers with risk of cardiovascular mortality were log-linear (all P ≤ 0.017 for trend) and did not differ according to diabetes status (all P ≥ 0.53 for interaction). After further adjustments for established risk factors, only CRP remained independently associated with cardiovascular mortality. HRs were 1.22 (1.12–1.32) per standard deviation higher log_e_ CRP concentration and 1.91 (1.50–2.43) when comparing individuals in the top versus bottom quartile. The associations of CRP, calprotectin, lactoferrin and neopterin with cardiovascular mortality did not differ by diabetes, suggesting that any potential prognostic value of these markers is independent of diabetes status.

## Introduction

Conventional risk factors fail to explain the variation in risk of cardiovascular disease (CVD). It is now widely accepted that CVD has a clear inflammatory component and that low-grade chronic inflammation can accelerate the formation and progression of atherosclerosis^1,2^ Accordingly, a substantial number of inflammatory biomarkers have been found to be linked to cardiovascular risk in general populations and therefore suggested to be novel risk markers that can aid cardiovascular risk prediction. However, some studies have indicated that the associations of inflammatory markers and cardiovascular risk differ according to subpopulations of risk profiles such as in individuals with diabetes^3–7^. The predictive ability of inflammatory markers in relation to cardiovascular risk may therefore vary and this should be taken into consideration if implementing them as markers to aid risk stratification. The accelerated atherosclerosis caused by chronic inflammation is thought to be driven partly by the altered functions and properties of immune cells such as macrophages and neutrophils^8,9^. Investigating biomarkers that reflect the activity of these immune cells and their roles in CVD is therefore of interest. Three markers were included in this study, namely neopterin, lactoferrin and calprotectin, along with C-reactive protein (CRP) for comparison.

Neopterin is a metabolite produced by activated macrophages. It is abundantly expressed within coronary lesions in patients with unstable angina pectoris^10^, and increases in blood of patients with coronary artery disease^11^. Studies show that increasing concentrations of neopterin can predict cardiac events in patients with chronic stable angina^12^ and acute coronary syndromes^13^, as well as fatal cardiac events in patients with diabetes^4^. Lactoferrin is a glycoprotein released from secondary granules of activated neutrophils, thought to have anti-atherogenic properties, such as inhibiting expression of endothelial adhesion molecules^14^ and cholesterol accumulation in macrophages^15^. Increasing lactoferrin concentrations correlate with the degree of coronary artery stenosis in patients undergoing angiography^16^ and are suggested to predict fatal cardiac events in patients with diabetes^3^. Calprotectin, also known as myeloid-related protein 8/-14 (MRP8/14) or S100A8/A9 complex, is an acute-phase protein, mainly released from neutrophils upon cell death or cellular activation^17,18^. Calprotectin has been shown not only to predict cardiovascular risk^19^, but is also suggested to be directly involved in the pathogenesis of atherosclerosis^20^. CRP, a general marker of inflammation, is extensively studied. Its association with CVD is well documented^21^, but a causal role of CRP in atherothrombosis has been refuted^22,23^.

We therefore aimed to assess the associations of these four inflammatory markers, i.e. CRP, calprotectin, neopterin and lactoferrin, with the risk of cardiovascular mortality and if the associations differ according to diabetes status. Secondarily, we aimed to evaluate whether calprotectin, neopterin and lactoferrin, suggested to directly reflect the activity of immune cells, were more strongly related to cardiovascular mortality than CRP.

## Methods

### Study design and population

The HUNT study is a large population-based cohort, consisting of four health surveys including more than 125,000 participants from the Nord-Trøndelag county of Norway^24^. Our study is based on data from the second survey (HUNT2; 1995–1997). All residents aged 20 years or older were invited to participate and in total, 65,237 attended in HUNT2 (69.5% of those invited, of which > 97% were of Caucasian origin). Data were collected from participants through questionnaires, clinical examinations and blood sampling, described in detail elsewhere^25^. Details of the study are available on the HUNT website (http://www.ntnu.no/hunt).

**Figure 1:**
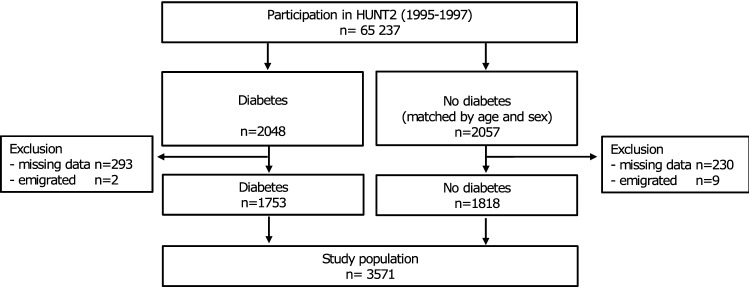
Inclusion of study participants.

We included all the participants in HUNT2 who were defined as having diabetes (n = 2,048) either by self-reports of a diabetes diagnosis (88%), self-reported use of anti-diabetic medication, or a non-fasting plasma glucose level ≥ 11.1 mmol/L (Fig. 1). Additionally, we included an equally sized random selection of age- and sex-matched participants without diabetes from the same survey. Analyses were restricted to individuals with complete information on the inflammatory markers of interest and potential confounding factors (i.e. hypertension, smoking, body mass index, blood lipids). Participants with missing data were less than 1–3%, except for smoking status for which data were missing in 9.3% and 8.6% of individuals with and without diabetes, respectively (Supplementary Table 1). Multiple imputation was not considered necessary because of the low proportion of participants with missing values. In total, 3,571 individuals (87%), of which 49% had diabetes, were followed up on cause of death.

### Follow up and outcome ascertainment

Individual person time at risk was calculated from the date of inclusion in HUNT2 (1995–97) until the date of death or end of follow up (December 31st, 2010). Participants (n = 11) who emigrated during follow-up were censored on the relevant date; otherwise there was no loss to follow-up. The Norwegian Cause of Death Registry provided information on the date and primary cause of death for all individuals. The primary outcome was death by CVD, as classified according to the International Classification of Disease code version 9 and 10 (ICD-9: 401–459.9; ICD-10: I10-99).

### Other study variables

Prevalent cardiovascular disease was defined by the self-reported history of myocardial infarction, angina pectoris or stroke. Self-reported smoking status was categorized into never, former or current smoking. Hypertension was defined as systolic blood pressure of ≥ 140 mmHg or diastolic blood pressure of ≥ 90 mmHg or use of antihypertensive medication. We calculated body mass index (BMI) as weight (kilograms) divided by the square of the height (meter), waist to hip-circumference ratio, and non-HDL cholesterol (the difference between the total cholesterol and high-density lipoprotein cholesterol). Non-fasting blood samples were collected at inclusion and serum concentrations of total cholesterol, HDL cholesterol, triglycerides, glucose and creatinine were analysed the same day by the available methods at that time^25^. Serum aliquots were stored at − 80 °C for later analysis of inflammatory markers. An in-house two-step ELISA (enzyme-linked immunosorbent assay) for immunological quantification of lactoferrin (LF) concentrations was used as previously described^26^, applying rabbit anti-human LF antibodies (Agilent, Santa Clara, CA, USA). ELISAs were also used to quantify neopterin (DRG Instruments, Marburg, Germany) and calprotectin (Bühlmann MRP8/14, Bühlmann Laboratories AG, Schönenbuch, Switzerland). CRP was measured using a high-sensitivity latex-enhanced immunoturbidimetric assay (Cardiophase hs-CRP, Siemens, Erlangen, Germany). For this assay the limit of quantitation of CRP was 0.16 mg/L. Because of volume restrictions, samples were pre-diluted 1:1 with NaCl 0.9%; implying that values less than 0.16 mg/L were assigned a value of 0.32 mg/L (a linearity by dilution experiment showed a mean recovery of 106% with the 95% confidence interval (CI) 104–108%).

### Statistical analyses

Descriptive data are given as means (standard deviation, SD), medians (interquartile range, IQR) or proportions (%). The Mann–Whitney *U*-test and *X*^2^ test were used to compare continuous and categorical variables, respectively. Statistical analyses were performed in the overall study population (adjusting for diabetes), and separately according to diabetes status. Quantile regression (due to non-normal distribution) was used to examine whether differences in median concentrations of inflammatory markers depended on age, gender or BMI. Spearman`s rho was calculated to assess the correlation between the four inflammatory markers.

We used Cox proportional hazard models to estimate hazard ratios (HRs) with 95% confidence intervals (CIs) for associations of the inflammatory markers with cardiovascular mortality. All estimated associations were adjusted for attained age (as the time scale) to ensure that participants were compared to other participants of the same age. The HRs were calculated within quartiles of the distributions (non-transformed concentrations), and per SD increase (log_e_ transformed concentrations) for each inflammatory marker. The SD for baseline log_e_ CRP concentration was 1.07, corresponding to approximately threefold higher non-transformed concentration (i.e. e˄1.07 = 2.92). Likewise, one SD increase in log_e_ calprotectin, log_e_ neopterin and log_e_ lactoferrin was approximately equivalent to a 1.7-fold, 1.9-fold and a 1.4-fold increase in their non-transformed concentrations.

Three models, where age was adjusted for by the time axis, were fitted for each inflammatory marker: Model 1 added sex and diabetes status, Model 2 added prevalent CVD, hypertension, BMI, smoking status, total cholesterol, HDL cholesterol, triglycerides and creatinine, and Model 3 added the three other inflammatory markers. Potential confounders were selected based on prior knowledge of known risk factors for CVD. The linearity of the associations was assessed across quartiles of the inflammatory markers with tests of trend. Statistical interaction between diabetes and the inflammatory markers was assessed by adding an interaction term to Model 1–3 for each marker.

The proportional hazards assumption was evaluated by the Schoenfeld test and log (−log) plots. P-values < 0.05 were considered statistically significant, and estimated associations were assessed by 95% CI.

We performed sensitivity analyses (based on Model 2) to account for potential effects of infections or ill health on baseline levels of inflammatory markers that may overestimate their associations with cardiovascular outcome. We did this by excluding participants with CRP > 10 mg/L, or participants with prevalent CVD.

Data were analysed using the software package STATA, (version 15.1, StataCorp, College Station, TX, USA).

### Ethics declarations

Written informed consent was obtained upon participation. The study protocol complies with the ethical guidelines of the Declaration of Helsinki and the present study was approved by the Norwegian Data Inspectorate and by the Regional Committee for Medical and Health Research Ethics (2017/1802).

## Results

In total, 1590 (44.5%) individuals died during a median follow-up of 13.9 years. Of these, 896 (56.4%) had diabetes at inclusion. 728 (20.4%) died from cardiovascular disease (CVD), of which 415 (57%) had diabetes at inclusion. Table 1 describes the distribution of baseline characteristics of participants of the study population according to diabetes status. CRP, neopterin and lactoferrin were higher among individuals with diabetes, also when adjusting for age, sex, and body mass index. The four inflammatory markers were weakly to moderately correlated (Spearman’s rho ≤ 0.61 for all combinations, p < 0.001, Supplementary Table 2).

**Table 1:** Baseline characteristics of the study population according to diabetes status.

Characteristic	No diabetes n = 1818	Diabetes n = 1753	P-value
Age (years)	68.2 (55.2–75.7)	68 (55.1–75.5)	0.74
Male sex, n (%)	938 (52)	918 (52)	0.64
Prevalent CVD, n (%)^a^	377 (21)	461 (27)	< 0.001
**Smoking, n (%)**			0.019
Never	765 (42)	802 (46)	
Former	596 (33)	575 (33)	
Current	457 (25)	376 (22)	
Hypertension, n (%)^b^	1215 (66)	1363 (78)	< 0.001
Systolic BP (mmHg)	148 ± 24	153 ± 24	< 0.001
Diastolic BP (mmHg)	84 ± 13	85 ± 14	0.32
Body mass index (kg/m^2^)	26.8 (26.5–29.0)	29 (25.5–31.7)	< 0.001
Waist-hip-ratio^c^	0.86 (0.80–0.92)	0.90 (0.84–0.95)	< 0.001
Non-fasting glucose (mmol/L)	5.4 (4.9–6.0)	9.4 (6.6–12.7)	< 0.001
Total cholesterol (mmol/L)	6.2 (5.5–7.1)	6.1 (5.2–7.1)	< 0.001
HDL cholesterol (mmol/L)	1.3 (1.1–1.6)	1.2 (1–1.4)	< 0.001
Non-HDL cholesterol (mmol/L)	4.9 (4.1–5.8)	4.8 (3.9–5.7)	0.24
Triglycerides (mmol/L)	1.61 (1.17–2.23)	2.12 (1.37–3.02)	< 0.001
Creatinine (µmol/L)	90 (81–99)	90 (81–101)	0.13
**Inflammatory markers**			
CRP (mg/L)	1.86 (0.95–3.88)	2.76 (1.32–5.67)	< 0.001
Calprotectin (mg/L)	2.93 (2.08–4.07)	2.93 (2.02–4.17)	0.97
Neopterin (nmol/L)	4.2 (2.9–6.6)	6.2 (4.4–8.4)	< 0.001
Lactoferrin (µg/L)	1004 (809–1195)	1037 (817–1215)	0.001

Data are given as medians (interquartile range), means ± standard deviation or proportions (%).

CVD Cardiovascular disease, BP Blood pressure.

^a^Self-reported history of myocardial infarction, angina pectoris or stroke.

^b^Systolic BP of ≥ 140 mmHg or diastolic BP of ≥ 90 mmHg or self-reported use of antihypertensive medication.

^c^Information on waist-hip-ratio was available in 3556 individuals (1740 with diabetes and 1816 without diabetes.

**Figure 2:**
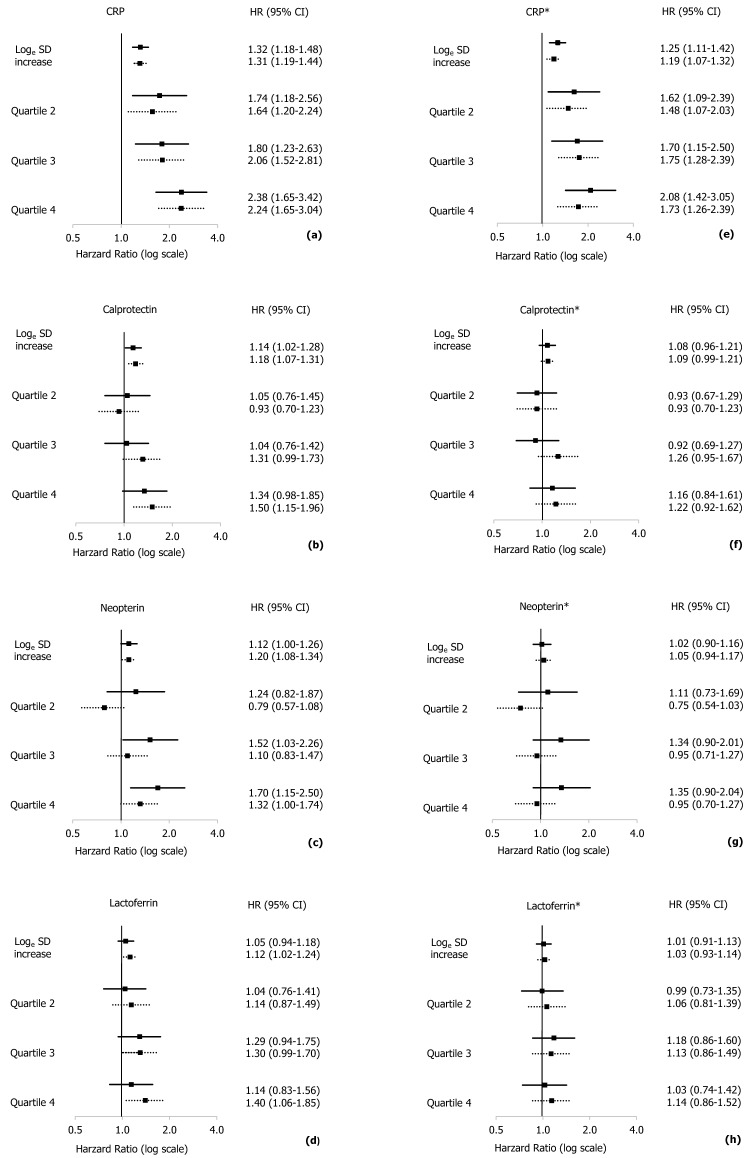
Inflammatory markers and risk of cardiovascular mortality according to diabetes status. *CI* Confidence interval, *HR* Hazard Ratio, *CVD* cardiovascular disease. HR and 95% CI computed per standard deviation (SD) log_e_ concentration of inflammatory markers and within quartiles of distributions (non-transformed values) separately for individuals without diabetes (solid lines) and with diabetes (dotted lines). Panel (**a**–**d**) Adjusted for age (time-axis) and sex. Panel (**e**–**h**)*: Additionally adjusted for prevalent CVD (self-reported history of myocardial infarction, angina pectoris or stroke), hypertension, smoking status, total cholesterol, HDL cholesterol, triglycerides, creatinine and body mass index (BMI).

Adjusted for age and sex, the estimated HR (95% CI) for cardiovascular mortality associated with diabetes was 1.83 (1.60–2.01). Additionally adjusted for established risk factors, the HR for individuals with diabetes was 1.70 (1.46–1.98), with insignificant changes when adding the inflammatory markers. All four inflammatory markers were positively and log-linearly associated with cardiovascular mortality (all P ≤ 0.017) after adjustment for age, sex and diabetes (Table 2). There were no significant statistical interactions between diabetes and the inflammatory markers, when adding this interaction term to the main models (all P ≥ 0.53 for interactions). When additional confounders were added, only CRP remained independently associated with cardiovascular mortality. Comparing individuals in the top quartile versus the bottom quartile, the HR was 1.91 (1.50–2.43), and per standard deviation (SD) increase in log_e_ CRP concentration the HR was 1.22 (1.12–1.32). The associations remained practically unchanged after additional mutual adjustments for the other three inflammatory markers. In separate analyses by diabetes status, the magnitude of the associations were comparable for individuals with and without diabetes with overlapping 95% confidence intervals (Fig. 2).

**Table 2:** Inflammatory markers and risk of cardiovascular mortality in overall study population.

Biomarker		No. of deaths	Model 1 HR (95% CI)	Model 2 HR (95% CI)	Model 3 HR (95% CI)
**CRP**					
Per log_e_ SD		728	1.32 (1.17–1.47)	1.22 (1.12–1.32)	1.21 (1.11–1.32)
*P* for interaction			0.94	0.94	0.94
Quartile 1	< 1.10 mg/L	99	Reference	Reference	Reference
Quartile 2	1.10–2.27 mg/L	188	1.69 (1.32–2.15)	1.54 (1.21–1.97)	1.55 (1.21–1.98)
Quartile 3	2.27–4.75 mg/L	189	1.73 (1.35–2.20)	1.47 (1.14–1.88)	1.48 (1.15–1.91)
Quartile 4	≥ 4.76 mg/L	252	2.41 (1.90–3.05)	1.91 (1.50–2.43)	1.88 (1.46–2.43)
*P* for trend			< 0.001	< 0.001	< 0.001
**Calprotectin**					
Per log_e_ SD		728	1.17 (1.08–1.26)	1.08 (1.00–1.16)	1.02 (0.92–1.13)
*P* for interaction			0.54	0.61	0.63
Quartile 1	< 2.05 mg/L	182	Reference	Reference	Reference
Quartile 2	2.05–2.93 mg/L	166	0.99 (0.80–1.22)	0.94 (0.76–1.16)	0.91 (0.73–1.13)
Quartile 3	2.93–4.11 mg/L	183	1.23 (1.00–1.52)	1.12 (0.91–1.38)	1.02 (0.81–1.29)
Quartile 4	≥ 4.11 mg/L	197	1.42 (1.15–1.74)	1.17 (0.94–1.44)	1.03 (0.79–1.34)
*P* for trend			< 0.001	0.073	0.63
**Neopterin**					
Per log_e_ SD		728	1.17 (1.08–1.27)	1.02 (0.95–1.12)	1.01 (0.93–1.10)
*P* for interaction			0.64	0.75	0.92
Quartile 1	< 3.4 nmol/L	107	Reference	Reference	Reference
Quartile 2	3.4–5.3 nmol/L	157	1.14 (0.89–1.46)	1.03 (0.80–1.32)	1.04 (0.81–1.33)
Quartile 3	5.3–7.6 nmol/L	192	1.23 (0.96–1.57)	1.04 (0.81–1.33)	1.04 (0.82–1.33)
Quartile 4	≥ 7.6 nmol/L	272	1.46 (1.15–1.84)	1.04 (0.81–1.33)	1.02 (0.79–1.30)
*P* for trend			0.0014	0.63	0.87
**Lactoferrin**					
Per log_e_ SD		728	1.09 (1.01–1.18)	1.02 (0.95–1.10)	0.97 (0.86–1.07)
*P* for interaction			0.53	0.63	0.57
Quartile 1	< 816 µg/L	190	Reference	Reference	Reference
Quartile 2	816–1022 µg/L	187	1.10 (0.90–1.35)	1.05 (0.85–1.28)	1.00 (0.81–1.24)
Quartile 3	1022–1785 µg/L	187	1.24 (1.02–1.52)	1.12 (0.91–1.37)	1.04 (0.83–1.30)
Quartile 4	≥ 1785 µg/L	164	1.26 (1.02–1.55)	1.07 (0.87–1.33)	0.94 (0.73–1.21)
*P* for trend			0.0170	0.45	0.71

*CI* Confidence interval, *HR* Hazard Ratio, *CVD* cardiovascular disease. Estimated HRs per standard deviation (SD) increase by baseline log_e_ concentrations, and within baseline concentrations (non-transformed) divided into quartiles. Model 1: Adjusted for age (as time scale), sex and diabetes. Model 2: Model 1 added prevalent CVD (self-reported history of myocardial infarction, angina pectoris or stroke), hypertension, smoking status, total cholesterol, HDL cholesterol, triglycerides, creatinine and body mass index (BMI). Model 3: Model 2 added the other 3 inflammatory markers.

P for interaction: statistical significance testing interaction between diabetes and each inflammatory marker. P for trend: statistical significance of linear trend across quartiles of each inflammatory marker.

In sensitivity analyses excluding participants with CRP > 10 mg/L (n = 354) the estimated associations remained similar. In analyses including only participants without prevalent CVD (n = 2733), the HRs per SD increase in log_e_ CRP and log _e_ calprotectin concentrations were 1.28 (1.15–1.41) and 1.14 (1.03–1.27), respectively. The results per SD increase in log_e_ neopterin and log_e_ lactoferrin were 1.06 (0.95–1.19) and 1.04 (0.95–1.15), respectively. There were still no significant statistical interactions of diabetes and the inflammatory markers (all P ≥ 0.37 for interaction).

## Discussion

In this prospective study, individuals with diabetes had almost twice as high risk of cardiovascular mortality as individuals without diabetes, after adjusting for traditional risk factors and inflammatory markers. The initial associations of calprotectin, neopterin and lactoferrin with cardiovascular mortality were no longer statistically significant after multivariable adjustments, suggesting that these inflammatory markers are not independent of traditional risk factors. In contrast, CRP remained independently associated with cardiovascular mortality, and to a similar magnitude as found by others^21,27^.

Importantly, the strength of the associations between the inflammatory markers and cardiovascular mortality did not differ by diabetes status. This is in contrast to other studies suggesting different prognostic accuracy of inflammatory markers in diabetes versus non-diabetic individuals^3–7^. Two studies found that neopterin and lactoferrin were predictors of ischemic heart disease in subjects with diabetes, but not in individuals without diabetes^3,4^. These studies, however, were small; statistical analyses were only done in separate groups according to diabetes status and interaction was apparently not formally tested. Three other studies found that CRP was a significant predictor of CVD only among individuals without diabetes^5–7^. In these studies, statistical interaction was apparently not formally tested. In contrast, two larger meta-analyses have demonstrated that the association between CRP and cardiovascular mortality was similar in individuals with and without diabetes^21,27^.

Established risk factors such as obesity, diabetes, hyperlipidemia, and smoking are all associated with increased inflammation^21^. Obesity causes chronic-low grade inflammation, with adipose tissue serving as a significant source of pro-inflammatory cytokines, including interleukin 6 (IL-6) which in turn induces production of C-reactive protein^28^. Higher levels of circulating calprotectin are positively correlated with obesity, smoking, LDL-cholesterol, and inversely with HDL cholesterol^29–31^. In a previous study, we found that serum calprotectin correlated with the neutrophil count (Spearman correlation coefficient 0.56) in a healthy population^32^. Studies show that leucocyte count, and neutrophils in particular, correlate with cardiovascular mortality^33^ and some suggest that calprotectin may be the link between neutrophil count, cardiovascular risk factors and CVD^31^. Excluding individuals with prevalent CVD in the sensitivity analysis, strengthened the associations of calprotectin with cardiovascular mortality risk. Studies investigating the pathophysiological mechanisms of calprotectin or its components have suggested an involvement in vascular inflammation and injury^34,35^, and in the promotion of atherogenesis in diabetes^36,37^. Further studies are needed to help shed light on a potential casual role of calprotectin in atherosclerosis.

A strength of this study was the relatively large study population, the long follow-up time, and the extensive registration of variables in the HUNT2 study, which enabled us to adjust for a range of possible confounders. The study design is unique, in including all individuals with diabetes from a large population cohort. In most prospective studies that include individuals with diabetes, these individuals constitute < 5–10% of the total study population^21,27^. We also included four biomarkers that could be related to different aspects of inflammation.

There were some limitations to our study. No clear distinction could be made between type 1 and type 2 diabetes, although a previous study from the same cohort found that ~ 80% were classified with type 2 diabetes^38^. Incomplete information regarding the duration of diabetes made it impossible to adjust for this factor in the primary analyses. Since we only had data on cardiovascular mortality and not on cardiovascular events, we could not assess associations of the inflammatory markers with overall cardiovascular risk. The stability of the inflammatory markers during long-term storage may be an issue of concern, considering the samples had been stored for about 20 years before analysis. Although CRP has been shown to remain highly stable in long-term stored serum^39^, less is known about the stability of the other three inflammatory markers. Nevertheless, all blood samples were stored under the same conditions, enabling comparison within the study of individuals with and without diabetes. Because the inflammation markers were only measured once at the beginning of follow-up, we could not examine the possible effects of time-dependent changes in the level of inflammation. However, studies show that CRP is as consistent within individuals over time, as total cholesterol and systolic blood pressure^40^. Residual confounding caused by unmeasured factors as medication use, lifestyle or socioeconomic factors may affect our estimates. Including individuals with prevalent CVD could introduce bias by reverse causality, even if this variable was adjusted.

During the last two decades, several inflammatory markers have been suggested as biomarkers that can aid cardiovascular risk stratification, but very few are actually used in clinical settings. In the search for useful inflammatory risk markers, the key cytokine interleukin-6 (IL-6) has proven to be a stronger predictor of CVD than its downstream marker CRP^41^. However, high-sensitivity CRP is a well-established, inexpensive analysis, measured routinely in many clinical laboratories. In contrast to IL-6, CRP has been proposed as a tool for individual cardiovascular risk assessment in several settings^42^. We did not have sufficient sample volumes to include analysis of IL-6.

In summary, our study showed that the associations of the tested inflammatory markers with cardiovascular mortality did not differ by diabetes status, suggesting that any potential prognostic value of these markers is the same in a sub-population of diabetes. Only CRP remained independently associated with cardiovascular mortality following multivariable adjustment.

## Supplementary Information

Supplementary Information.

## Data Availability

Data from the HUNT Study are available upon reasonable request from the HUNT Research Centre (https://www.ntnu.edu/hunt/data), following approval from the Regional Research Ethics Committee. However, restrictions apply to the availability of the data for the present paper, which were used under license for the current study and are not publicly available, in accordance with Norwegian law.
