# Supplementary material for: Inflammatory markers and risk of cardiovascular mortality in relation to diabetes status in the HUNT study
Source: Sci Rep. 2021 Aug 2;11:15644. doi: 10.1038/s41598-021-94995-8 (PMC8329190; doi:10.1038/s41598-021-94995-8)
Supplement: Supplementary file 1 — Supplementary Information. [file 41598_2021_94995_MOESM1_ESM.pdf]

Inflammatory markers and risk of cardiovascular mortality in relation to diabetes status in the HUNT study

Lena Løfblad, Gunhild Garmo Hov, Arne Åsberg and Vibeke Videm

Supplementary Table 1

Baseline characteristics of participants with missing data versus final study population

Characteristic	Excluded due to missing data		Study population	P-value
	n		n=3751	
Age (years)	0	74.1 (66.2-80.2)	68.1 (55.1-75.6)	<0.001
Female sex, n (%)	0	356 (67%)	1715 (48%)	<0.001
Prevalent CVD, n (%) [1]	41(1%)	136 (27%)	838 (24%)	<0.001
Smoking, n (%)	369 (9%)			<0.001
Never		101 (61%)	1668 (45%)	
Former		42 (26%)	1213 (33%)	
Current		22 (13%)	855 (25%)	
Hypertension, n (%) [2]	21 (0.5%)	421 (79%)	2578 (72%)	<0.001
Systolic BP (mmHg)	21 (0.5%)	159 ± 25	151 ± 24	<0.001
Diastolic BP (mmHg)	21 (0.5%)	86 ± 14	84 ± 13	0.002
Body mass index (kg/m^2)	91 (2.2%)	27.3 (24.7-31.1)	27.5 (24.7-30.4)	0.17
Waist-hip-ratio [3]	62 (1.5%)	0.87 (0.82-0.92)	0.88 (0.82-0.93)	0.03
Non-fasting glucose (mmol/L)	10 (0.2%)	6.3 (5.3-9.3)	6.1 (5.2-9.4)	0.12
Total cholesterol (mmol/L)	10 (0.2%)	6.5 (5.6-7.4)	6.1 (5.3-7.1)	<0.001
HDL cholesterol (mmol/L)	14 (0.3%)	1.2 (1-1.5)	1.2 (1.0-1.5)	0.53
Non-HDL cholesterol (mmol/L)	14 (0.3%)	5.2 (4.3-6.0)	4.8 (4.0-5.7)	<0.001
Triglycerides (mmol/L)	11 (0.3%)	1.96 (1.40-2.84)	1.80 (1.25-2.64)	0.001
Creatinine (µmol/L)	11 (0.3%)	89 (81-101)	90 (81-100)	0.99
**Inflammatory markers**				
CRP (mg/L)	3 (0.1%)	2.48 (1.21-5.37)	2.27 (1.10-4.75)	0.07
Calprotectin (mg/L)	3 (0.1%)	2.66 (1.81-3.97)	2.93 (2.05-4.11)	0.002
Neopterin (nmol/L)	6 (0.2%)	6.7 (4.7-9.9)	5.3 (3.4-7.6)	<0.001
Lactoferrin (µg/L)	20 (0.5%)	984 (759-1190)	1022 (817-1215)	0.01

Data are given as medians (interquartile range), means ± standard deviation or proportions (%).

CVD Cardiovascular disease, BP Blood pressure.

[1] Self-reported history of myocardial infarction, angina pectoris or stroke. [2] Systolic BP of ≥ 140 mmHg or diastolic BP of ≥ 90 mmHg or self-reported use of antihypertensive medication.

[3] Information on waist-hip-ratio was available in 3556 individuals of the total study population.

Supplementary Table 2

Spearman`s correlation coefficients between inflammatory markers

**Inflammatory markers**	CRP	Calprotectin	Neopterin
Calprotectin	0.30*	-	
Neopterin	0.23*	0.06*	-
Lactoferrin	0.23*	0.61*	0.07*

Asterisk indicates level of statistical significance: *p< 0.001
